# Identification of Microbiome Etiology Associated With Drug Resistance in Pleural Empyema

**DOI:** 10.3389/fcimb.2021.637018

**Published:** 2021-03-16

**Authors:** Zhaoyan Chen, Hang Cheng, Zhao Cai, Qingjun Wei, Jinlong Li, Jinhua Liang, Wenshu Zhang, Zhijian Yu, Dongjing Liu, Lei Liu, Zhenqiang Zhang, Ke Wang, Liang Yang

**Affiliations:** ^1^ Intensive Care Unit, The First Affiliated Hospital of Guangxi Medical University, Nanning, China; ^2^ School of Medicine, Southern University of Science and Technology, Shenzhen, China; ^3^ Department of Orthopedic Trauma and Hand Surgery, The First Affiliated Hospital of Guangxi Medical University, Nanning, China; ^4^ Pulmonary and Critical Care Medicine, The First Affiliated Hospital of Guangxi Medical University, Nanning, China; ^5^ Department of Infectious Diseases and Shenzhen Key Laboratory for Endogenous Infection, Shenzhen Nanshan People’s Hospital of Shenzhen University, Shenzhen, China; ^6^ National Clinical Research Center for Infectious Diseases, Shenzhen Third People’s Hospital, Southern University of Science and Technology, Shenzhen, China; ^7^ Department of Respiratory and Critical Care Medicine, Liuzhou People’s Hospital, Liuzhou, China

**Keywords:** empyema, metagenomic, microbiome, resistome, community structure, *Staphylococcus aureus*

## Abstract

Identification of the offending organism and appropriate antimicrobial therapy are crucial for treating empyema. Diagnosis of empyema is largely obscured by the conventional bacterial cultivation and PCR process that has relatively low sensitivity, leading to limited understanding of the etiopathogenesis, microbiology, and role of antibiotics in the pleural cavity. To expand our understanding of its pathophysiology, we have carried out a metagenomic snapshot of the pleural effusion from 45 empyema patients by Illumina sequencing platform to assess its taxonomic, and antibiotic resistome structure. Our results showed that the variation of microbiota in the pleural effusion is generally stratified, not continuous. There are two distinct microbiome clusters observed in the forty-five samples: HA-SA type and LA-SA type. The categorization is mostly driven by species composition: HA-SA type is marked by *Staphylococcus aureus* as the core species, with other enriched 6 bacteria and 3 fungi, forming a low diversity and highly stable microbial community; whereas the LA-SA type has a more diverse microbial community with a distinct set of bacterial species that are assumed to be the oral origin. The microbial community does not shape the dominant antibiotic resistance classes which were common in the two types, while the increase of microbial diversity was correlated with the increase in antibiotic resistance genes. The existence of well-balanced microbial symbiotic states might respond differently to pathogen colonization and drug intake. This study provides a deeper understanding of the pathobiology of pleural empyema and suggests that potential resistance genes may hinder the antimicrobial therapy of empyema.

## Introduction

Empyema is defined as the presence of germs and/or macroscopic pus in the pleural cavity, which is a serious infection with high rates of morbidity and mortality (Asai et al., 2017). Previous analysis of pleural effusion microbiome of empyema patients was mainly based on bacterial cultivation (Lasken and McLean, 2014), PCR and Multiplex bacterial PCR (Blaschke et al., 2011; Franchetti et al., 2020). Recently, microbial characterization of empyema was conducted using targeted 16S rRNA metagenomic analysis (Dyrhovden et al., 2019). However, the increase in the complexity of the pathogens and the usage of antibiotic pre-treatment can reduce the sensitivity of the conventional bacterial cultivation (Le Monnier et al., 2006); PCR-based analysis is highly dependent on the design and availability of primers and thus has very low throughput; 16S rRNA amplicon-based metagenomic analysis has a limitation in detecting microbiome at the species level, and may introduce PCR-biases that mask the true community composition (Brooks et al., 2015).

Next-generation sequencing (NGS)-based metagenomic approach has been employed to examine the population structures and functions of the microbiome in human and environmental samples, which provides biomarkers and risk assessment information, such as antibiotic-resistant bacteria and antibiotic-resistance genes (ARGs) (Cote et al., 2016). In this study, we collected pleural effusion (PE) samples from 45 empyema patients and applied NGS metagenomic analysis to characterize the microbial community and antibiotic resistome. We identified two distinct microbial communities in pleural effusion samples, where *Staphylococcus aureus* serves as a biomarker. Furthermore, the abundance of antibiotic resistance genes is correlated with microbial diversity. Our study reveals the potential risks of treatment failure of pleural empyema due to the high abundance of ARGs in the microbial community and provides data for better understanding of the pathophysiological mechanism in empyema.

## Materials and Methods

### Ethics Statement

The research was approved by the Ethical Review Committee of the First Affiliated Hospital of GuangXi Medical University [Approval Number: 2017(KY-E-078)], and filed with the Ethical Committee of Southern University of Science and Technology [Approval Number: 20200090].

### Definition of Pleural Empyema and Samples Collection

A pleural empyema is defined as pus (macroscopic purulence) in the pleural space or pleural fluid with organisms present on Gram stain or culture, pleural fluid pH <7.20 or pleural fluid glucose <60 mg/dL with clinical evidence of infection (Light, 2006). 45 empyema patients involved in this study were recruited in the First Affiliated Hospital of GuangXi Medical University from June 2017 to May 2019. The non-repetitive pleural effusion (PE) samples were collected during thoracentesis, transported in a low-temperature transport box, and stored at -80 °C until further processing.

### DNA Isolation

DNA of the samples was obtained by mechanical disruption of bacterial cells using the SeptiFast Lysis kit (Roche, Mannheim, Germany) on a MagNALyzer^®^ instrument (Roche Diagnostics GmbH, Mannheim, Germany) followed by DNA extraction and purification on a MagNA Pure compact automated extractor (Roche, Mannheim, Germany). DNA quality and potential contamination were checked on 1% agarose gel. DNA concentration and purity were checked using NanoPhotometer^®^ spectrophotometer (IMPLEN, CA, USA).

### DNA-seq Library Construction and Sequencing

Illumina sequencing libraries were prepared with 500ng gDNA template for each sample according to the TruSeq DNA Sample Preparation Guide (Illumina, 15026486 Rev.C). Concentrations of the constructed libraries were measured using Qubit 2.0 and diluted to 1 ng/μL. Agilent 2100 Bioanalyzer and Bio-RAD CFX 96 Real-Time PCR System (use Bio-RAD KIT iQ SYBR GRN) were used to qualify and quantify the sample libraries (library effective concentration >10nM). The qualified libraries were then sequenced on Illumina Hiseq 2500 platform with 150 bp paired-end reads (Anoroad, Beijing, China).

### Metagenomic Analysis

The raw reads generated from samples (11.5~21.5 GB) were trimmed and filtered to remove low quality (Q ≤ 20) and short reads (length < 50 bp) using Trimmomatic (version 0.39) (Bolger et al., 2014). Reads aligned to the human genome (hg38, Genome Reference Consortium Human Reference 38) were removed (identity cutoff ≥ 90%; maximum mismatches, 10 bp) by Bowtie2 (version 2.3.5.1) (Langmead and Salzberg, 2012). Clean Metagenomic sequences were assembled using the MEGAHIT (version 1.2.9) with default parameters (Li et al., 2016) (**Supplemental Table S1**). The open reading frames (ORFs) prediction was then conducted for assembled contigs using Prokka program (version 1.12) (Seemann, 2014). CD-HIT (version 1.12) was used to cluster genes from each sample based on the parameters (BLASTn identities > 95%, coverage > 90%) (Fu et al., 2012). We aligned high-quality reads against the gene catalog using Salmon v1.2.1 (identity cutoff ≥ 95%) and calculated the corresponding relative abundance of each gene (Patro et al., 2017). ARGs were predicted by mapping the metagenomes to the Comprehensive Antibiotic Resistance Database (CARD) database with 80% identities (Alcock et al., 2020). The taxonomic composition was performed using Kraken 2 software (Wood et al., 2019) based on NR databases. To detect the potential biomarkers, the linear discriminant analysis (LDA) effect size (LEfSe) method was used based on a normalized relative abundance matrix (Segata et al., 2011).

### Statistical Analysis and Network Analysis

Averages and standard deviations were computed using the base function in R 3.6.2. Venn diagrams were drawn with the Venn Diagram package, while heatmaps were generated using the pheatmap package by R 3.6.2. The α-diversity based on Shannon index on the species and ARGs profile in each sample was calculated to evaluate the species and ARGs diversities by R 3.6.2. Principal Coordinates Analysis (PCoA) was plotted based on Bray-Curtis dissimilarity to compare the species composition and ARGs profiles of the samples on R 3.6.2 in the vegan package. Correlation between microbial composition and resistome was calculated by pairwise Spearman’s rank correlation with coefficient > 0.80 and FDR adjusted *P* value <0.01. Co-occurrence network analysis was conducted in R platform with Hmisc and igraph package, and visualized by Gephy 0.9.2.

### Availability of Data and Materials

All data generated during this study are available at the Sequence Read Archive (SRA) under BioProject accession number PRJNA657096.

## Results

### Clinical Characteristics

45 participants were enrolled in the present study. The clinical characteristics and medication history of the individuals who participated in this study are summarized in **Table 1** and **Supplemental Table S2**. The laboratory bacterial culture showed that 12 (26.7%) were positive for culture only. Out of these, nine samples were of monomicrobial infection caused by *Nocardia farcinica* (PE2), *Klebsiella pneumoniae* (PE4), *Mycobacterium* (acid-fast bacilli) (PE8, PE16), *Candida albicans* (PE23), *Klebsiella oxytoca* (PE24), *Streptococcus constellatus* (PE30), *Escherichia coli* (PE41), and *Acinetobacter baumannii* (PE43). Another three samples had a mixed infection caused by *Candida albicans* and *Stenotrophomonas maltophilia* (PE28), *Candida tropical* and *Pseudomonas aeruginosa* (PE31), *Enterococcus faecium* and *P. aeruginosa* (PE40), respectively.

**Table 1:** Basic characteristics of the study participants with pleural effusion.

	*n* = 45*
Age, y	50.3 ± 19.4
Male	40 (88.9)
Signs and symptoms	
Pneumonia	26 (57.8)
Diabetes mellitus	10 (12.8)
Hypertension	7 (15.5)
Post-traumatic empyema infection	9 (20)
Tuberculous empyema infection	6 (13.3)
Hospital-acquired empyema infections	7 (15.5)
Anti-infective therapy before sampling	
Performed	41 (91.1)
Antibiotics	41 (91.1)
Anti-tuberculosis	8 (17.8)
Anti-fungal	2 (4.4)
Blood parameters	
Performed	45 (100)
Leucocytes (×10^9^/L)	15.3 ± 7.8
Neutrophils (×10^9^/L)	12.7 ± 7.5
C-reactive protein (mg/L)	124.6 ± 76.9
Pleural fluid parameters	
Performed	43 (95.6)
Protein (g/L)	42.1 ± 21.5
Glucose (mmol/L)	2.1 ± 3.7
Lactate dehydrogenase (U/L)	2816.9 ± 2300.1
Adenosine deaminase (U/L)	115.6 ± 92.5
Specimen collection time	
2017	12 (26.7)
2018	19 (42.2)
2019	14 (31.1)

*Mean (standard deviation), n (%).

### Hierarchical Clustering of the Pleural Empyema Microbia

**Figure 1:**
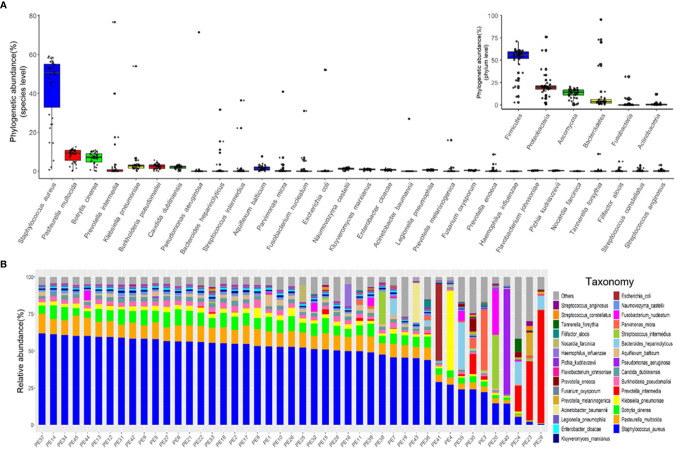
The major microbiome taxa at the phylum and species levels in the pleural effusion samples. **(A)** Box plot of species abundance variation for the 30 most abundant species as determined by read abundance. Species are colored by their respective phylum (see inset for color key). Inset displays the box plot of abundances at the phylum level. **(B)** Stacked bar plot of the 30 most abundant species in the pleural effusion microbiome.

We characterized the phylogenetic variation across the sequenced samples at the species and phylum levels. The 30 most abundant species (belonged to the six most abundant phyla) in empyema patients are shown in **Figure 1A**. The phylogenetic composition of the sequenced samples confirms that bacteria predominated in all samples and contributed more to phylogenetic diversity than eukaryotes and archaea. The phyla, *Firmicutes*, *Proteobacteria*, *Ascomycota* and *Bacteroidetes*, constitute the vast majority of the dominant pleural effusion microbiota. *Staphylococcus aureus*, *Pasteurella multocida*, *Botrytis cinerea*, *K. pneumoniae*, *Prevotella intermedia*, *Burkholderia pseudomallei* and *Candida dubliniensis* were identified to be the enriched species in empyema patients. Among which, *S. aureus* was the most abundant species across the samples analyzed.

Microbes in the pleural effusion undergo selective pressure from the host as well as from microbial competitors. This typically leads to the homeostasis of the ecosystem in which some species occur in high and many in low abundance (**Figure 1B**). *S. aureus* was the most variable species across samples, agreeing with relative abundance varied dramatically from 0.79% to 61.97%. In 36 samples, *S. aureus* was the most abundant species, with relative abundance ranging from 24.06% to 61.97% (24.06% in PE 30 sample, and more than 40% in other 35 samples). Meanwhile, the dominating species varied extensively across the other 9 samples (the relative abundance of *S. aureus* ranging from 0.79% to 29.04%), which were *Parvimonas micra* (PE3), *K. pneumoniae* (PE4), *Streptococcus intermedius* (PE20), *P. intermedia* (PE23, PE24, PE29), *Bacteroides heparinolyticus* (PE35), *P. aeruginosa* (PE40), and *E. coli* (PE41), respectively.

### Comparison of Microbial Community Composition of HA/LA-SA Group

**Figure 2:**
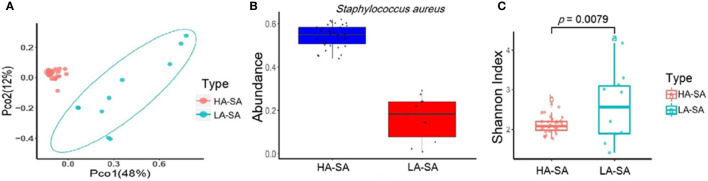
Microbiome composition of the HA-SA and LA-SA type samples. **(A)** Principal Coordinate Analysis. Species among the microbial community for each sample is generated based on the Bray-Curtis similarity matrix in HA- and LA-SA type. The first two components (PCo1 and PCo2) of the PCoA plot explained 48% and 12% variations, respectively, in two groups, with a wider range of within-group distribution in the LA-SA group. **(B)** The abundance levels of the main contributing species of two microbiome types. **(C)** Alpha diversity estimation. Significant differences in Shannon diversity estimates of microbial communities on species level in HA- and LA-SA types.

The within-sample (alpha) diversity (Shannon index) and the between-sample (beta) diversity (Principle Coordination Analysis, PCoA) were used to estimate the richness and composition of pleural effusion microbial species. PCoA based on the Bray-Curtis distance revealed that the samples formed two distinct clusters which can be differentiated by the variation in the level of the most abundant species, *S. aureus* (**Figure 2B**). We designate the two clusters as high abundance *S. aureus* type (HA-SA type, 35/45) and low abundance *S. aureus* type (LA-SA type, 10/45) (**Figure 2A**). As described above, *S. aureus* was the most enriched species with a relative abundance of more than 40% in all 35 samples of the HA-SA type, whereas each sample had distinctive dominating species (except the sample PE) with the relative abundance of *S. aureus* below 30% in the LA-SA type. Meanwhile, the alpha diversity of the LA-SA type was much higher than that of the HA-SA type (*P* = 7.91 × 10^−3^, Wilcoxon rank-sum test, **Figure 2C**).

In addition, diversity analysis was also performed to investigate the potential effects of different variables on the composition of the pleural effusion microbiota. Among the 45 samples, pneumonia, diabetes, hypertension, post-traumatic empyema infection, tuberculous empyema infection, hospital-acquired empyema infections, and specimen collection time had no significant effect on the microbiome composition (**Supplemental Figure S1**).

### Variation of Microbiome and Biomarkers Between HA/LA-SA Type

**Figure 3:**
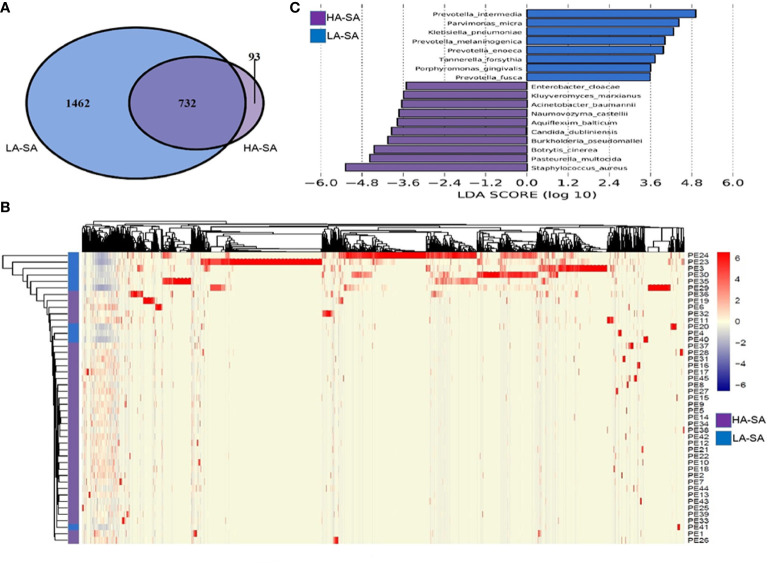
Comparison of pleural effusion microbiome composition among the HA- and LA-SA types. **(A)** Venn diagram showing the number of shared and unique species in the HA- and LA-SA group. **(B)** Heat map of the microbiome species composition for all samples. The abundance of each species was clustered to represent a heatmap. **(C)** Differentially abundant species were identified using linear discriminant analysis (LDA) coupled with effect size measurements (LEfSe). The cutoff value of the linear LDA was ≥3.5.

To determine the phylogenetic variation of the HA- and LA-SA types, we investigated in detail their differences in composition at the species level. Of the total 2287 detected species, 825 (36.1%) species were identified in HA-SA type while 2194 (95.9%) species were identified in LA-SA type. This was consistent with the results of alpha diversity. There were 732 species shared between the two types, accounting for 22.9% (732/2287) of the total number of species detected. The proportions of the shared species in the two types were 88.7% (732/825) in HA-SA type and 33.4% (732/2287) in LA-SA type, respectively (**Figure 3A**). Structure of the microbial community of the HA-SA type was relatively stable with high inter-sample consistency in the microbial compositions and the enriched species. In contrast, samples in LA-SA type had distinct microbial communities, and the microbiome observed showed no clear clustering (**Figure 3B**).

We screened key biomarkers (*i.e.*, key community members) using the LEfSe method to explore the distinctive microbial species in two types associated with empyema infection. Based on the selection criteria of LDA score of more than 3.5, we identified 18 microbial species as the key discriminants (**Figure 3C**). Ten species including *S. aureus*, *P. multocida*, *B. cinerea*, *Aquiflexum balticum*, *B. pseudomallei*, *C. dubliniensis*, *Naumovozyma castellii*, *Enterobacter cloacae*, *Kluyveromyces marxianus*, and *A. baumannii* were identified as key biomarkers in HA-SA group. Eight species including *Prevotella* spp. (including 4 species), *P. micra*, *K. pneumoniae*, *Tannerella forsythia*, and *Porphyromonas gingivalis* were significantly enriched in the LA-SA group.

### Abundant Antibiotic Resistome With Variation Between HA/LA-SA Type

**Figure 4:**
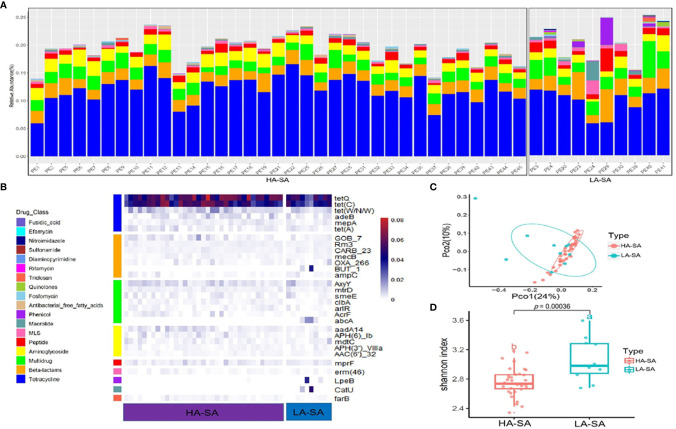
Abundant ARGs in the pleural effusion microbiome. **(A)** Stacked bar plot of antibiotics resistant classes in the pleural effusion metagenome. **(B)** The 30 most abundant ARGs in the HA- and LA-SA type were displayed by heatmap. **(C)** Principal Coordinate Analysis. ARG composition is independent of microbiome community composition. **(D)** Diversity estimates of detected resistance features. Significant differences in Shannon diversity estimates of different resistance features of the CARD database inside the HA- and LA-SA type.

Due to the distinct microbial community profiles of the HA-SA and LA-SA types, we further analyzed the antibiotic resistance genes (ARGs) in the pleural effusion samples. A total of 238 ARGs belonging to 18 ARG classes were detected across the samples. LA-SA type harbored all the 18 ARG classes and HA-SA type harbored 16 ARG classes (**Figure 4A**). Nonetheless, the dominant ARG classes in the LA-SA type were in common with that of the HA-SA type’s resistome, which is also in agreement with PCoA observations (**Figure 4C**). The abundant ARGs belonging to 6 dominant ARG classes, such as *tetQ* and *tetC* (coding for the tetracycline resistance), Gob-7 beta-lactams resistance gene, *axyY* (multidrug resistance), *aadA14* (aminoglycoside resistance), *mprf* (peptide resistance), *erm46* (MLS resistance) were predominant in both types (**Figure 4B**).

To better understand the influence of HA/LA-SA type on the ARGs in pleural effusion samples, Shannon α-diversity indices for HA/LA-SA type resistome were calculated. These results indicated that higher diversity was observed in the LA-SA type compared to the HA-SA type (**Figure 4D**). Several abundant ARGs detected in the LA-SA type such as *but-1*, *abcA*, *lpeB* and *catU* were undetected in the HA-SA type (**Figure 4B**).

**Figure 5:**
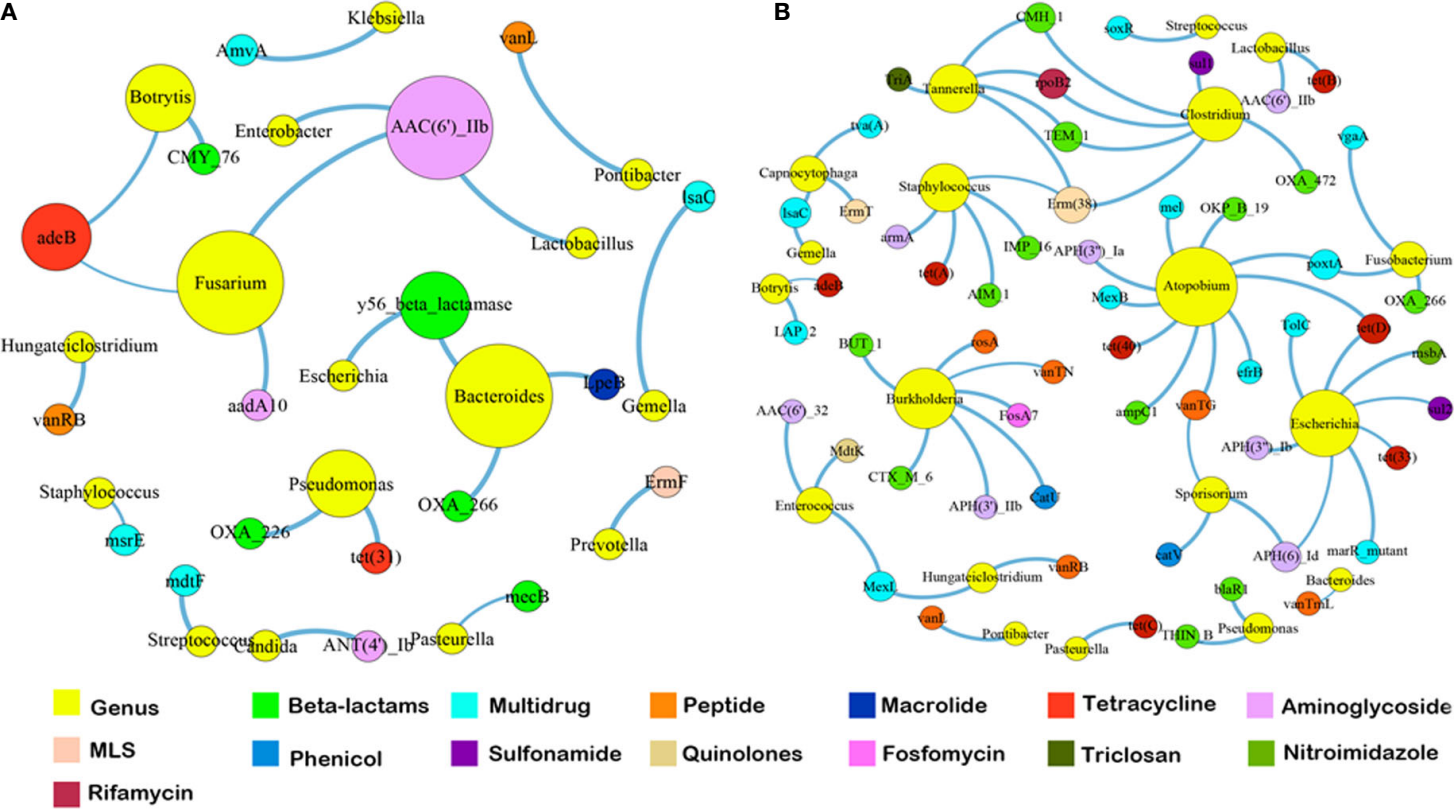
The co-occurrence networks among ARGs and bacterial genera in HA-SA type **(A)** and LA-SA type **(B)**. In the graph, each dot represents a kind of ARG or bacterial genera. The nodes are colored yellow represent bacterial genus; others colored according to ARG types. The size of each node is proportional to the number of its connections (degree). Each line (edge) represents the co-occurrence of two objects. Edge width is proportional to Spearman’s ρ value.

Since the dominant ARG classes were not correlated with microbial composition, we investigated the co-occurrence patterns between ARGs and microbial genera in the HA/LA-SA group using network analysis approach. In this study, if the ARGs and the co-existed microbial taxa possessed the significantly similar abundance trends among the different samples (Spearman’s *ρ* >0.8, *P*-value <0.01), one of the reasonable explanations of the corresponding similar abundance trends was because of some specific microbial taxa carrying some specific ARGs, which has been verified by Forsberg’s study (Forsberg et al., 2014). The co-occurrence network in the HA-SA type was comprised of 34 nodes and 22 edges (**Figure 5A**), two bacterial genera were speculated as possible major hosts of ARGs: *Bacteroides* was strongly correlated with macrolide resistance gene (*lpeB*) and beta-lactams resistance genes (*bla*_OXA-266_ and y56-beta-lactamase gene), whereas *Fusarium* was strongly correlated with aminoglycoside resistance genes (*aac*(6)-IIb and *aadA10*) and tetracycline resistance gene (*adeB*). A more complicated co-occurrence network, comprising of 74 nodes and 66 edges, was observed from the LA-SA type (**Figure 5B**). Six bacterial genera, including *Atopobium*, *Burkholderia*, *Escherichia*, *Clostridium*, *Staphylococcus* and *Tannerella*, were strongly correlated with various ARGs. Especially, *Staphylococcus* in the LA-SA type, was the host of aminoglycoside resistance gene (*armA*), beta-lactams resistance genes (*bla*_AIM-1_ and *bla*_IMP-16_), MLS resistance gene (*erm38*) and tetracycline resistance gene (*tet A*).

## Discussion

In this study, the microbiome and resistome of pleural effusion collected from 45 empyema infection patients were explored based on NGS metagenomic analysis.

Previous studies showed that the geographical location of infection is closely related to the expected pathogenic bacteria, and *S. aureus* is the most preponderant species in sub-tropical areas (Hassan et al., 2019). In our study, *S. aureus* was detected in all of the pleural effusion samples and was the most abundant species in the HA-SA type. This suggests that the result of metagenomic analysis follows the geographical pattern and aligns with the findings of previous studies. On the other hand, we also detected other dominant species, which together with *S. aureus* to construct a complex microbiome in pleural effusion. Upon the change in the abundance of *S. aureus* in the microbial communities, we observed two significantly different microbiome compositions among samples, being the HA-SA type and LA-SA type. Virtually, none of the measured host properties, namely pneumonia, diabetes, hypertension, post-traumatic empyema infection, tuberculous empyema infection, hospital-acquired empyema infection and specimen collection time, significantly correlates with the microbiome types.

In the HA-SA type with the relative abundance of *S. aureus* more than 40%, we identified a highly similar and stable core microbial composition with low diversity. Such core microbial community consists of *S. aureus* as core species and 9 other enriched species, including 6 bacteria (*P. multocida*, *A. balticum*, *B. pseudomallei*, *E. cloacae*, *K. marxianus*, and *A. baumannii*) and 3 fungi (*B. cinerea*, *C. dubliniensis*, and *N. castellii*). It was reported that bacteria can thrive in both pleural fluid and pleural tissue (Popowicz et al., 2017). However, the invading mechanisms of microorganisms in the pleural cavity and features of the pathogenesis, such as the role of biofilm formation, have not yet been fully understood (Thomas et al., 2020). A recent study had reported that *P. aeruginosa* can form biofilm in an empyema model (Zhang et al., 2020). Some studies have also pointed out that fungi contribute to biofilm formation while *Candida app.* is the most common fungal flora in biofilm infection (Ramage et al., 2009). *S. aureus* and fungi have synergism in biofilm formation (Boase et al., 2011). In the HA-SA type, the co-existence of *S. aureus* and 3 fungi (including one *Candida* spp.) perhaps contributed to the formation of biofilm which would stabilize the microbiome community and help pathogens to escape from host immune clearance and increase antibiotic resistance (De Rudder et al., 2018).

In comparison, the LA-SA type with the relative abundance of *S. aureus* below 30% had a more diversified microbiome. The microbiome had several significantly enriched biomarkers including *Prevotella* spp. (including 4 species), *P. micra*, *K. pneumoniae*, *T. forsythia*, and *P. gingivalis*, which except for *K. pneumoniae* were anaerobic bacteria. Furthermore, these anaerobic bacteria are involved in various oral infections, especially associated with periodontal infections. Some studies have already found a remarkably high involvement of anaerobic oral bacteria in empyema infection, whereas odontogenic infections have been identified as a potential risk factor of empyema (Kobashi et al., 2008). The specificity of geographical areas should be emphasized here, the oral microbiome varies across locations and may influence the bacterial composition of pleural empyema (Dyrhovden et al., 2019). The exact mechanisms whereby oral flora gain access to the pleural space are incompletely understood. However, it is speculated that facultative and anaerobic oral bacteria, able to spread *via* deoxygenated venous blood, which is a possible infection route of oral bacterial pleurisy (Dyrhovden et al., 2019).

The positive rate of routine pleural effusion culture of the collected samples was not high, only 26.7%. Standard pleural effusion cultures are usually positive in approximately 20-40% of cases (Menzies et al., 2011). This is likely to be a result of the combination of prior antibiotic treatment, low bacterial concentration in pleural effusion and possibly causal agents that are difficult to be isolated in the laboratory due to stringent requirements. In the HA-SA type, the culture-positive rate was 17.14% (6/35), without any *S. aureus* culture positive results. According to previous reports, the *S. aureus* culture positivity are different depending on whether an empyema is community-acquired or healthcare-acquired. The positive rate is 12% in community-acquired empyema and 20% in Hospital-acquired empyema, respectively (Koma et al., 2017; Brims et al., 2019). We might raise the hypothesis that the *S. aureus* culture negativity in this study may reflect the adaptation of bacteria to a specific host niche, such as *S. aureus* to biofilm, and the niche conditions could not be replicated by the routine *in vitro* culture conditions resulting in negative culturing results (Pommepuy et al., 1996; Lowder et al., 2000; Oliver, 2010). In the LA-SA type, the culture-positive rate was as high as 60% (6/10), and culture-positive pathogens in 3 samples matched with the most abundant species identified by metagenomic analysis. There were not any anaerobic bacteria cultured. The most compelling evidence for “occult” anaerobes in empyema fluid is detection of bacterial DNA or RNA using massive parallel sequencing. This approach identified anaerobic bacteria in 70% patients with empyema and no known etiology (Cobo et al., 2018).

The LA-SA type and HA-SA type possess common ARG classes which the most dominant ARG classes were tetracycline resistance and beta-lactam resistance. On one hand, the microbes growing in pleural effusion and pleural tissue would be selected by antibiotics treatment (91.1%, 41/45), especially beta-lactams antibiotics (39, 86.7%), which is frequently acquired *via* horizontal gene transfer (HGT). Previous research reported that tetracycline resistance genes are often integrated into mobile genetic elements (MGE), and prevalent in a large number of microbial populations colonized in human oral cavity and intestinal tract (Seville et al., 2009). These bacterial populations, functioning as the repository of tetracycline-resistant genes, contribute to the spread of ARGs into pathogenic bacteria through different mechanisms such as HGT, without prior treatment of tetracycline antibiotics. All of the above reasons may lead to the highly similarity in the resistomes of the HA/LA-SA type. Meanwhile, our study showed a higher diversity of ARGs in the samples with increased microbial diversity. It is possible that biofilm formation may contribute to the increase in the antibiotic resistance of the HA-SA type and reduce the dependence on ARGs. The observation that *S. aureus* solely correlating with multidrug resistance gene (*msrE*) in the HA-SA type while a more complex network existing between *S. aureus* and more diverse ARGs in the LA-SA group may support this inference.

In conclusion, the variation of microbiota in the pleural effusion is generally stratified, not continuous. *S. aureus* plays an important role in the shaping the microbial structures. Microbial community does not shape the resistomic profiles of the two types, which possess common ARG classes. The higher the microbial diversity, the more diverse the ARG profiles. These findings highlighted the capacity and advantage of NGS metagenomics for investigating the empyema and pathophysiological mechanisms to provide better understanding of the disease.

## Data Availability Statement

The datasets presented in this study can be found in online repositories. The names of the repository/repositories and accession number(s) can be found in the article/**Supplementary Material**.

## Ethics Statement

The studies involving human participants were reviewed and approved by the ethical review committee of the First Affiliated Hospital of GuangXi Medical University [Approval Number: 2017(KY-E-078)], and the Ethical Committee of Southern University of Science and Technology [Approval Number: 20200090]. The patients/participants provided their written informed consent to participate in this study.

## Author Contributions

LY and KW conceived the study and participated in data analysis and discussion. ZYC, HC, ZC, QW, JLL, JHL, WZ, ZY, DL, LL, and ZZ carried out the experiments, analyzed the data, and drafted the manuscript. All authors contributed to the article and approved the submitted version.

## Funding

This work was supported by the National Natural Science Foundation of China under Grant [number 81760024]; Guangdong Natural Science Foundation for Distinguished Young Scholar under Grant [number 2020B1515020003]; the Southern University of Science and Technology (SUSTech) to LY under Start-up Grants [number Y01416206]; the Medical Excellence Award Funded by the Creative Research Development Grant from the First Affiliated Hospital of Guangxi Medical University.

## Conflict of Interest

The authors declare that the research was conducted in the absence of any commercial or financial relationships that could be construed as a potential conflict of interest.
