# Supplementary figures and images for: Identification of Microbiome Etiology Associated With Drug Resistance in Pleural Empyema
Source: Front Cell Infect Microbiol. 2021 Mar 16;11:637018. doi: 10.3389/fcimb.2021.637018 (PMC8008065; doi:10.3389/fcimb.2021.637018)

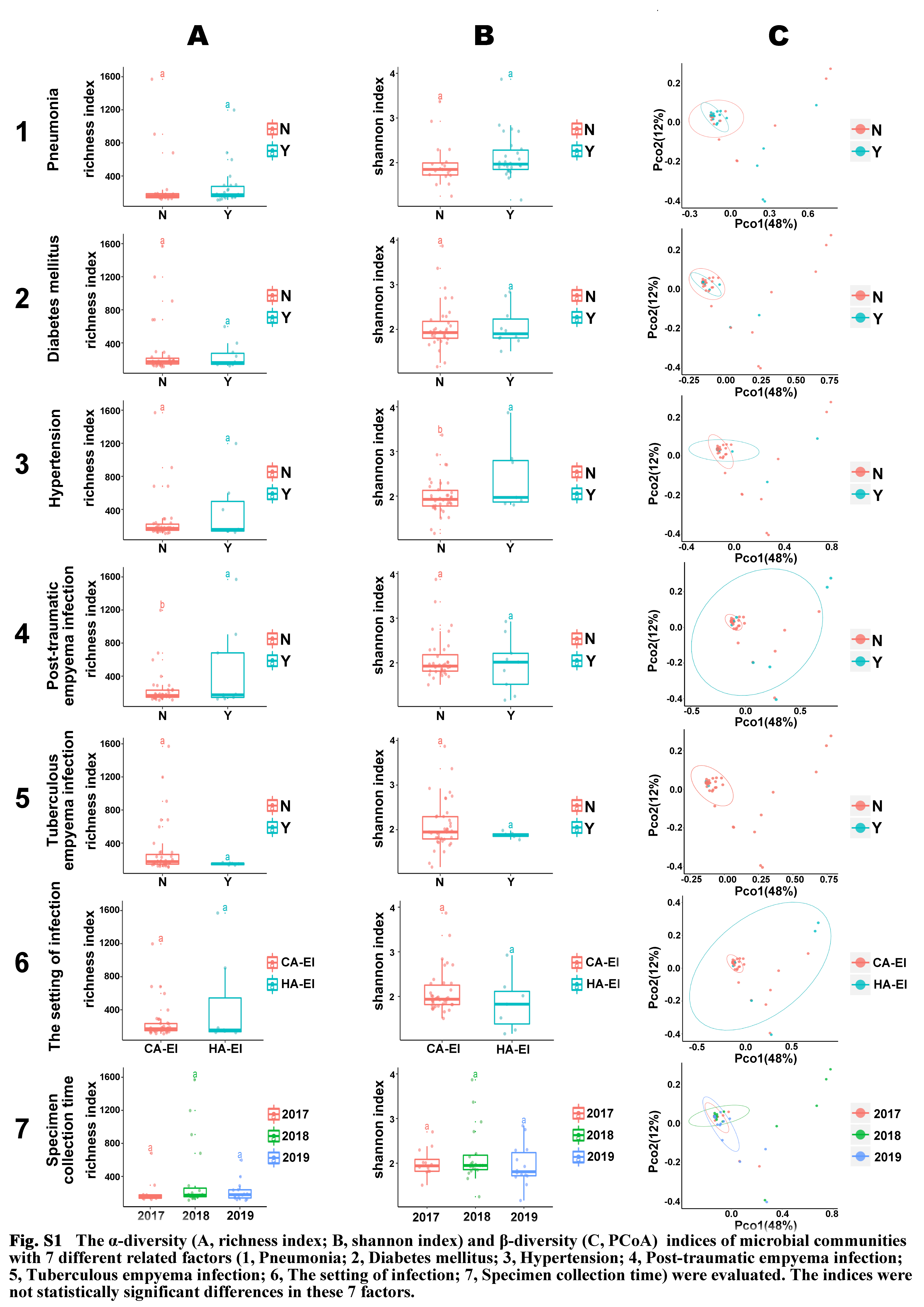

Supplement: Supplementary file 1 [file Image_1.tif]
